# Modular prosthesis fracture in a patient with developmental dysplasia of the hip: a case report and literature review

**DOI:** 10.1186/s12891-021-04325-2

**Published:** 2021-05-14

**Authors:** Yuan-Pei Cheng, Xiao-Kang Cheng, Yong-Bo Li, Qian-Ru Zhang, Hao Feng, Yi-Han Zhong, Yan-Bo Zhang, Han Wu

**Affiliations:** 1 Department of Orthopaedics, China-Japan Union Hospital of Jilin University, Jilin, 130033 China; 2 Department of Orthopaedics, Affiliated Hospital of Chengde Medical University, Hebei, 067000 China; 3 Shanghai Jiao Tong University School of Medicine, Shanghai, 200025 China

**Keywords:** Total hip arthroplasty, Developmental dysplasia of the hip, Prosthesis fracture, Nonunion, Case report

## Abstract

**Background:**

Modular prosthesis fracture, especially distal femoral fracture, is a rare complication of total hip arthroplasty (THA). However, it is catastrophic, and may have a serious impact on the patients. A distal femoral prosthesis fracture in a patient with developmental dysplasia of the hip (DDH) with nonunion at the subtrochanteric osteotomy site has not yet been reported in any literature. This report presents the first such case, with a purpose of analyzing the causes of modular prosthesis fractures and nonunion of the osteotomy area, so as to provide orthopedic surgeons with experience and lessons.

**Case presentation:**

We report the case of a 52-year-old woman with the distal femoral prosthesis fracture after THA and subtrochanteric osteotomy for Crowe type IV DDH. The patient had severe pain in the left thigh and her activities were limited. Plain radiographs revealed fracture of the left distal femoral prosthesis and nonunion in the subtrochanteric osteotomy region of the left femur. After a revision of the THA, the patient’s symptoms were resolved.

**Conclusions:**

A prosthesis fracture combined with nonunion at the subtrochanteric osteotomy site is a rare complication. Modular THA combined with a subtrochanteric osteotomy in the treatment of Crowe type IV DDH should reduce the damage to blood supply and avoid further nonunion of the osteotomy area, which may otherwise lead to modular prosthesis fractures. A detailed preoperative plan and suitable rehabilitation program may help minimize the occurrence of subtrochanteric osteotomy nonunion and reduce complications, including femoral prosthesis fractures, in patients with DDH.

## Background

Developmental dysplasia of the hip (DDH) is a condition in which structural deformities of the acetabulum, proximal femur, and joint capsule result in joint instability, ultimately leading to hip joint dislocation. While the cause of DDH is not well elucidated, it is thought to be related to genetics, breech presentation, and other risk factors. DDH can be diagnosed on the basis of the femoral head shape, acetabular index, acetabular coverage, and concentricity [1]. Early DDH is treated by closed reduction with dynamic and static splinting [2], while advanced DDH is treated by open reduction with Salter osteotomy [1]. The Sivash-Range of Motion (S-ROM) modular hip prosthesis has become more popular over the past few decades [3]. Modular hip prostheses allow for the adjustment of the limb length, femoral anteversion, and femoral prosthesis offset [4]. Modular total hip arthroplasty (THA) combined with a subtrochanteric osteotomy is commonly used in the treatment of Crowe type IV DDH. Fractures of modular femoral prostheses of THA are rare, and the incidence is estimated to be very low [5]. According to literature reports, prosthesis fractures are usually located at the head-neck and stem-sleeve junctions [6, 7]. There are few reports of fractures of the distal stem of a modular prosthesis. Nonunion of the osteotomy area is a complication of subtrochanteric osteotomy, and its incidence is very low. This is the first report on a femoral prosthesis fracture in a patient with DDH with nonunion at the subtrochanteric osteotomy site. The present case study aims to discuss the causes of modular prosthesis fractures and nonunion of the osteotomy area, so as to provide data that guide orthopedic surgeons.

## Case presentation

**Fig. 1:**
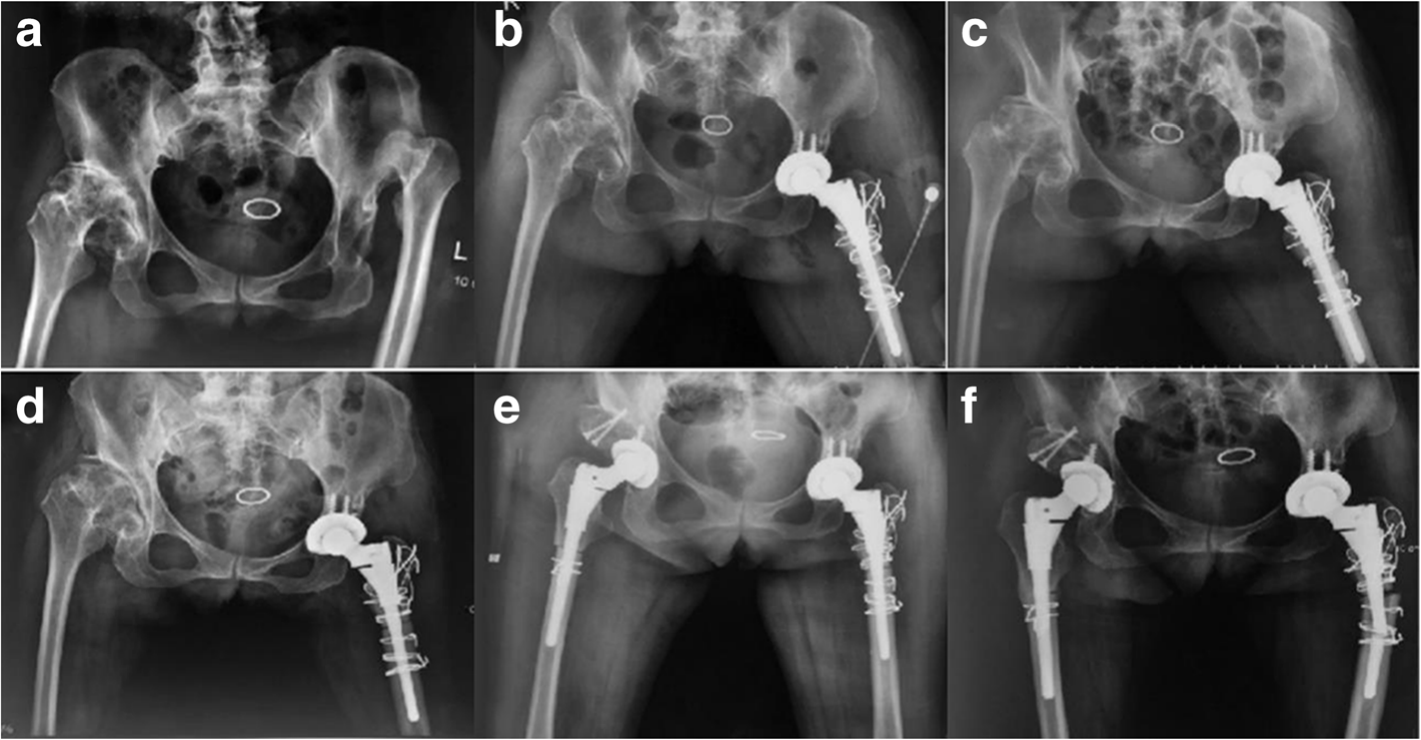
Radiographic images before revision surgery (**a-f**). **a** Crowe type IV DDH was found at the left hip before primary surgery. **b**-**e** Radiographic images of the patient’s left primary THA 1 day, 1 month, 3 months, 5 months postoperatively. **f** The radiographic examination of the patient’s left primary THA 14 months postoperatively shows that the distal stem is broken and the subtrochanteric osteotomy of the left femur is not healed

**Fig. 2:**
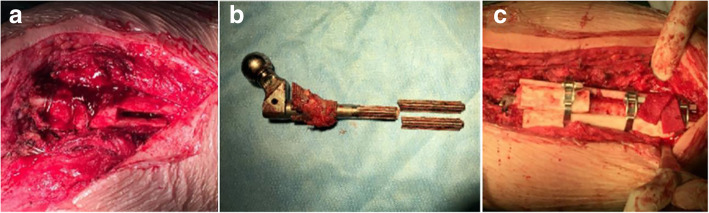
Intraoperative radiographs (**a-c**). **a** The left distal femur was fenestrated. **b** The fractured prosthesis was removed. **c** The proximal femoral bone defect was reconstructed using a cortical bone plate

**Fig. 3:**
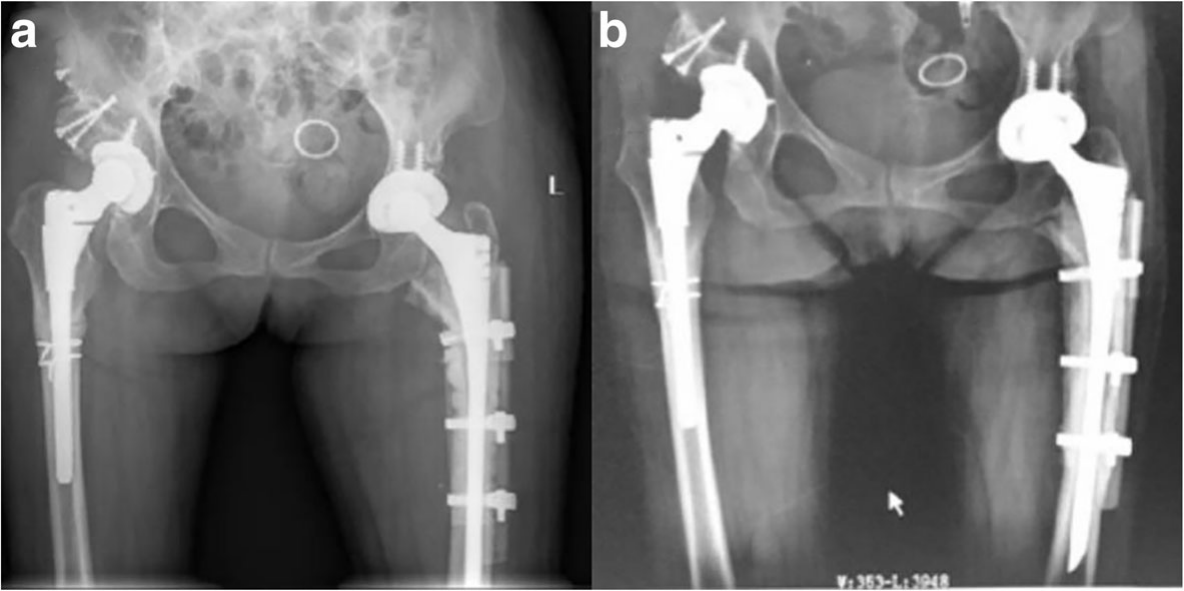
Radiographic images after revision surgery (**a-b**). **a** Radiograph of the patient’s left revision THA 1 day postoperatively showed that the prosthesis was fixed. **b** Radiographic image showed that the subtrochanteric osteotomy of the left femur is healed at a 12-month follow-up after the patient’s revision surgery

A 52-year-old woman with bilateral DDH (Fig. 1a) underwent left and right THA with subtrochanteric osteotomy at a local hospital in November 2016 and February 2017, respectively. The bilateral stem was a modular hip system (Just company, China, imitation of S-ROM). The patient felt no pain in the hip and performed normal activities after the operation. Postoperative follow-up X-ray examination revealed that the osteotomy area of the left femur had not healed (Fig. 1b–e). No further treatment was administered. The patient denied a family genetic history. In October 2017, the patient experienced spontaneous left-sided thigh pain when bearing weight. She was very frightened, and could neither stand on both legs or walk. The movement of the left hip joint was limited, and the patient could not participate in social activities or even care for herself. Therefore, she came to our hospital for treatment. Physical examination revealed tenderness of the left thigh and a limited movement of the left hip joint (0–90° in extension-flexion). A radiographic image showed a fracture in the distal part of the stem and revealed that the subtrochanteric osteotomy site of the left femur had not healed (Fig. 1f). Laboratory examination was not suggestive of an infection (white blood cell count: 4.52 × 10^9^/L; erythrocyte sedimentation rate: 6 mm/h; C-reactive protein level: 6.26 mg/L; neutrophil percentage: 64.9%). She was diagnosed with a modular prosthesis fracture, and we performed a revision surgery. The acetabular component was not revised, because a preoperative X-ray examination and intraoperative findings revealed a well-fixed acetabular component. The proximal stem and sleeve were removed using an extractor and osteotomes, and the bone growth of the removed sleeve was found to be good. The fractured prosthesis was removed using the window technique (Fig. 2a–b). A fully-coated revision stem (200 mm in length, 9 mm in diameter; Chung-Li company, China) was implanted after reduction of the fracture, and the proximal femoral bone defect was reconstructed using a cortical bone plate (Xin-Kang-Chen company, China) (Fig. 2c). The catheter was removed 24 h after the operation. Antibiotics were injected intravenously for 3 days after the revision surgery, while anticoagulants were administered orally until 35 days after the operation. Following the principle of moving from easy and passive exercises to difficult and active exercises, the patient performed muscle strength and joint mobility exercises for the left limb in the early stages and hip function and gait exercises in the later stages. Partial weight-bearing was allowed 7 days after the operation, and completely weight-bearing was allowed 4 weeks after the operation. The patient was followed up via outpatient examinations. During the follow-up period, the patient’s condition gradually improved. One year postoperatively, the patient felt no pain and could walk normally. The left hip joint movement was not restricted. The patient could take care of herself, and was able to perform all of her activities of daily living. The visual analogue scale score and the Harris Hip score significantly improved from 7 and 48 preoperatively to 2 and 85 1 year postoperatively, respectively. The patient was very satisfied with the therapeutic effect. Plain radiographs showed union in the subtrochanteric osteotomy site of the left femur (Fig. 3).

## Discussion and conclusions

The treatment of DDH with THA is challenging due to acetabular dysplasia [8], femoral deformities [9], soft tissue abnormalities [10], and biomechanical changes [11]. Modular hip prosthesis has achieved good clinical results in the treatment of DDH due to its flexibility [12]. Sometimes, a subtrochanteric osteotomy is also necessary in the treatment of DDH [13], as it provides better torsion stability of the femoral stem, preserves the proximal femur bone, balances limb length, overcomes soft tissue contractures, and reduces the risk of sciatic nerve paralysis [14]. Modular THA combined with a subtrochanteric osteotomy for patients with Crowe type IV DDH has achieved favorable long-term results [15]. However, there are some complications of modular THA combined with subtrochanteric osteotomy, such as postoperative dislocations, prosthesis fractures, nerve palsies, a postoperative residual limp, and nonunions [8, 16, 17]. Modular femoral prosthesis fracture following THA has a low incidence rate. Furthermore, the incidence of nonunion at the subtrochanteric osteotomy site ranges from 0 to 6% [18, 19]. According to literature reports, this is the first report of a femoral prosthesis fracture in a patient with DDH with nonunion at the subtrochanteric osteotomy site.

The causes of modular prosthesis fractures following THA include fretting, corrosion, a thin diameter of the distal stem, high offset, fatigue stress, and obesity according to other cases. Waly et al. [20] described a 64-year-old patient who underwent THA for DDH. Seven years after the operation, she suddenly felt pain in her right hip, which resulted in a fall. An X-ray examination revealed a fracture of the femoral prosthesis. During the revision, the synovial fluid was found to be black and the synovial tissue was stained with metal. There was no evidence of loosening of the femoral stem or the femoral sleeve. The case indicates that mechanical micromotion and crevice corrosion at the stem-sleeve interface can lead to a prosthesis fracture. Norman et al. [21] described two patients with modular prosthesis fractures. The first patient in their report was a 69-year-old who suddenly felt pain and could not stand. X-ray examination revealed an obvious fracture and displacement of the prosthesis. The second patient in their report was a 70-year-old who gradually suffered pain and had difficulty in walking. X-ray examination revealed no obvious fracture of the modular prosthesis. Failure analysis concluded that initiation and propagation of a fatigue crack in the modular interface could be the cause of the fractures, which eventually led to corrosion-fatigue failure. The thin diameter of the distal stem in an obese or active patient may also be a possible cause of prosthesis fracture. Ellman et al. [22] described a 59-year-old patient who underwent a right THA due to Ficat stage IV avascular necrosis of the right femoral head. Five years after the operation, he suffered persistent pain in the right hip. X-ray examination revealed a fracture of the modular femoral neck, indicating that fretting, corrosion, and failure can cause modular prosthesis fractures. McNabb et al. [23] described a 66-year-old female who underwent a right THA for degenerative osteoarthritis. Three years after the operation, the patient fell. X-ray examination revealed no fracture or implant failure. Seven years postoperatively, another X-ray examination revealed a fracture of the modular femoral stem prosthesis, despite the patient reporting no pain or functional problems. The case demonstrated that multiple bending moments in a small diameter stem could lead to a fracture of the modular femoral stem prosthesis at the coronal slot. Wright et al. [5] described a 49-year-old patient who underwent a left THA for degenerative osteoarthritis and who fell 4 years after the operation, but did not experience any pain. Two months after the fall, the patient bent down and heard a click, and was not able to stand. X-ray examination revealed good fixation of the acetabular and femoral components and a fracture of the modular neck. In this case, the modular neck fracture was found to have been caused by fretting and corrosion due to the height and weight of the patient and the long varus neck of the prosthesis. Uchiyama et al. [24] described a 46-year-old patient who underwent a left THA for DDH. Three years and 8 months after the operation, he experienced a sudden left groin pain and was unable to bear weight on the left side. X-ray examination revealed a fracture of the modular neck, indicating that stress-induced fractures of the modular neck may be caused by a high-offset and a small modular component in obese and active patients. Pearce et al. [25] described two patients with spontaneous fractures of their modular prostheses. The first patient was a 75-year-old who experienced a sudden pain in her right thigh, and the second patient was a 68-year-old who experienced a sudden pain in her left thigh and knee. Neither patient suffered any trauma. The two cases indicated that fatigue stress could lead to fractures of modular femoral prostheses.

The causes of nonunion at the subtrochanteric osteotomy site include peeling of the periosteum and damage to the blood supply at the osteotomy site due to excessive steel wire fixation during the initial revision [26]. Insufficient rotational stability [27], a high temperature of the subtrochanteric osteotomy [8, 15], and premature load or improper activity may also affect the healing of the osteotomy site.

Some studies have seemingly explained the relationship between prosthesis fracture and nonunion. Benoist et al. [28] reported prosthesis fractures and nonunion of the osteotomy area following revision THA. Nonunion of the subtrochanteric osteotomy area after THA significantly increases the stress of the prosthesis, which can thereby increase the risk of prosthesis fracture. A study by Crowninshield et al. [29] revealed that the mechanical stress at the junction could increase by 92% in case of nonunion of the trochanteric osteotomy.

However, there are certain limitations to our report. First, biomechanical evaluation of the fractured prostheses was not performed. Second, the follow-up period was short. Third, the number of cases, with just one patient analyzed, was small. Further studies with large samples and long-term follow-ups are still required to test the conclusion as follows.

In conclusion, modular femoral prosthesis fracture combined with a nonunion at the subtrochanteric osteotomy site is a very rare complication after THA and subtrochanteric osteotomy for Crowe type IV DDH. The respective causes of modular prosthesis fractures and nonunion at the osteotomy site are multifactorial. In the current case, nonunion was caused by a destruction of the blood supply to the osteotomy area due to excessive steel wire fixation based on the present case. Poor rotational stability, fretting, and fatigue stress secondary to nonunion eventually resulted in a distal femoral prosthesis fracture according to other cases. To reduce the incidence of subtrochanteric osteotomy nonunion, which may in turn help prevent complications such as prosthesis fractures, orthopedic surgeons should make detailed preoperative plans encompassing the use of preoperative plain radiographs to assess the limb length, femoral anteversion, and femoral prosthesis offset and ensure appropriate intraoperative selection of the prosthesis, reduction of steel wire fixation to avoid excessive disruption of the blood supply, and an appropriate rehabilitation program (such as weight loss) to avoid premature weight bearing and promote a reduction of inappropriate activities.

## Data Availability

The datasets used and/or analyzed during the current study are available from the corresponding author on reasonable request.

## Abbreviations

THA: Total hip arthroplasty
DDH: Developmental dysplasia of the hip
S-ROM: Sivash-range of motion
